# Mycobacterial Diseases of Animals

**DOI:** 10.4061/2011/292469

**Published:** 2011-11-30

**Authors:** Mitchell V. Palmer, Michael D. Welsh, Jesse M. Hostetter

**Affiliations:** ^1^National Animal Disease Center, Agricultural Research Service, USDA, Ames, IA, USA; ^2^Veterinary Sciences Division, Agri-Food and Biosciences Institute, Belfast BT4 3SD, UK; ^3^College of Veterinary Medicine, Iowa State University, Ames, IA 50010, USA

Although *Mycobacterium tuberculosis* and *Mycobacterium leprae* are the most notable mycobacterial human pathogens, *Mycobacterium bovis*, *Mycobacterium avium* subsp. *avium*, *Mycobacterium avium* subsp. *paratuberculosis*, *Mycobacterium ulcerans*, and other mycobacteria are the etiology of important diseases in humans and a wide range of animal species including, cattle, sheep, goats, deer, possums, badgers, elephants, dogs, cats, birds, amphibians, and fish. Moreover, species such as *M. bovis *represent serious zoonotic pathogens and have become important agents at the interface of humans, domestic livestock, and wildlife. 

This special issue on mycobacterial diseases of animals contains 26 papers comprising 6 reviews, 3 case reports, and 17 original research papers on various topics including animal models, immunology, epidemiology, microbiology, pathology, environment, and history. Authors from 13 different countries provide a diverse examination of mixed topics. The first 6 papers are reviews; M. Munyeme and H. M. Munang'andu discuss numerous anthropogenic factors that impact wildlife, livestock, and humans in the habitat of the endangered lechwe antelope. Bovine tuberculosis eradication efforts are impeded by the presence of an established wildlife reservoir of *M. bovis*. Accordingly, M.V. Cunha et al. describe the current status of bovine tuberculosis in Portugal, discussing interspecies transmission and the impact of infected wildlife on the status of tuberculosis in cattle. Many mycobacterioses occur in nonmammalian hosts and K. Dhama et al. provide an in-depth review of avian mycobacterioses.

 In many species, pathogenic mycobacteria undergo a prolonged asymptomatic, or latent period, after which disease is reactivated in a subset of infected hosts. Containment of disease and latency likely coincide with shifts in host immune response. Accordingly, B. L. Plattner and J. M. Hostetter thoroughly review the role of gamma/delta T-lymphocytes in mycobacterial diseases of humans, cattle, and mice.

The last 2 reviews are historical in nature. First, M. Good and A. Duignan discuss tuberculin, the mainstay of TB testing. Their review emphasizes the origins, properties, limitations, and use of tuberculin in control programs, leading to the final review in which M. V. Palmer and W. R. Waters describe the genesis of the US bovine tuberculosis eradication effort in 1917. The authors describe laudable research conducted by veterinarians and other scientists, decades before an eradication program existed. 

The next 3 papers are best described as case reports, discussing mycobacterioses in wildlife. M. Pate et al. provide the first description of *Mycobacterium celatum-*induced disease in heretofore-unrecognized hosts in Slovenia. W. R. Waters and colleagues describe herds of fallow deer and elk with unusually high disease prevalence. The authors demonstrate the apparent misdiagnosis of numerous *M. bovis*-infected deer and elk and show the usefulness of novel serology based diagnostic tests. M. Carstensen and M. W. DonCarlos detail the uncovering of *M. bovis* in deer and cattle in Minnesota and their efforts to identify deer to cattle transmission, determine the prevalence of disease in free-ranging deer, and methods used to prevent the establishment of a persistent wildlife reservoir. 

Animal models of tuberculosis are extremely useful, particularly when conservation or ethics prevent experimental infection of the host of interest. This special issue's original research papers begin with L. McCallan et al.'s thorough description of ferrets infected with *M. bovis* via aerosol as a model for evaluation of tuberculosis vaccines. Research papers continue with a focus on epidemiology. S. Barandiaran et al. reminds us of often-overlooked hosts of bovine tuberculosis such as swine, examining transmission of *M. bovis* from cattle to swine using spoligotyping. Disease transmission between livestock and wildlife is of great interest. The next 2 papers focus on that interface. C. C. Okafor et al. examine deer to cattle transmission of *M. bovis* in northern Michigan. From Canada, T. K. Shury and D. Bergeson describe the use of various diagnostic strategies, as well as lesion distribution, and epidemiology of tuberculosis in elk and white-tailed deer in southwestern Manitoba. 

 Switching pathogens, host, and geography, A. A. Rita et al. examine the prevalence of ovine paratuberculosis in Italy using both serology and fecal culture. The suggested link between *Mycobacterium avium* subsp. *paratuberculosis* (*Map*) and Crohn's disease raises questions of public health significance. To that end, H. Okura and colleagues document a low prevalence of *Map* in muscle from *Map*-infected cattle, suggesting a bacteremic phase in bovine paratuberculosis. Emphasizing the broad host range of the myriad mycobacterial species, L. Durnez and colleagues discuss the presence of various mycobacteria in insectivores and rodents on cattle farms in Tanzania and compare findings to mycobacteria isolated from cattle.

Both *M. bovis* and *Map* are known to persist in the environment, which facilitates interspecies and intraspecies transmission. As a result, A. E. Fine et al. detail the survivability of *M. bovis* on various feedstuffs and organic matter. Prolonged survival in soil is a feature of *Map*. However, E. A. Raizman and collaborators show that not only can *Map* survive in soil, but also can pass through soil to ground water potentially finding its way to local watersheds. For researchers of paratuberculosis, the survivability of samples in the laboratory is important. E. A. Raizman et al. examine the effect of prolonged frozen storage on *Map* viability. 

Great effort and immense resources have been expended to study the diagnosis of mycobacterial diseases. Further complicating diagnosis can be concurrent infection by multiple mycobacteria in the same host. Using a gamma interferon release assay, C. Barry et al. examine the cell-mediated immune response of cattle experimentally infected with both *Map* and *Mycobacterium avium* subsp. *avium*. It is believed that exposure to nontuberculosis mycobacteria, such as *Map,* can result in falsely positive tuberculin skin test reactions. Consequently, S. D. Fitzgerald et al. document a low number of false-positive tuberculin test reactors in Michigan among cattle with confirmed *Map *infection. J. A. Fernández-Silva et al. use molecular epidemiological tools to characterize *Map* isolates from cattle in Colombia, while F. Delgado et al. describe the use of in situ PCR for detection of *Map* in formalin-fixed samples.

The host range of *M. bovis* includes most mammals including wild species such as African Cape Buffalo. Tuberculosis in buffalo on public and private lands is of concern not only from an animal health and conservation perspective, but also from an economic perspective due to the negative impact on ecotourism. Consequently, H. M. Munang'andu et al. describe the use of tuberculin testing in the formation of a tuberculosis-free herd of buffalo in Zambia's Kafue Basin.

Diagnosis of mycobacteriosis is accomplished either by confirming the pathogen's presence or examination of host immune response to the pathogen. Investigation of host immune response is foundational to the development of novel diagnostics. A. Jolly et al. examine the effects of antibodies, induced by the highly immunogenic lipoarabinomannan of *Map,* on phagocytosis and pathogen survival in bovine macrophages.

In humans, (multidrug resistant) MDR and (extensively drug resistant) XDR strains of *M. tuberculosis* are of serious concern. Emphasizing the zoonotic nature of *M. bovis*, S. D. Fitzgerald and colleagues examine *M. bovis* isolates from deer in the endemic region of Michigan for evidence of drug resistance similar to MDR and XDR patterns seen in *M. tuberculosis.*

